# Application of Artificial Intelligence Modeling Technology Based on Fluid Biopsy to Diagnose Alzheimer’s Disease

**DOI:** 10.3389/fnagi.2021.768229

**Published:** 2021-12-03

**Authors:** Yuan Sh, Benliang Liu, Jianhu Zhang, Ying Zhou, Zhiyuan Hu, Xiuli Zhang

**Affiliations:** ^1^Fujian Provincial Key Laboratory of Brain Aging and Neurodegenerative Diseases, School of Basic Medical Sciences, Fujian Medical University, Fuzhou, China; ^2^China National Center for Bioinformation, Beijing, China; ^3^Key Laboratory of Genomic and Precision Medicine, Beijing Institute of Genomics, Chinese Academy of Sciences, Beijing, China; ^4^Chinese Academy of Sciences Key Laboratory of Standardization and Measurement for Nanotechnology, Chinese Academy of Sciences Key Laboratory for Biomedical Effects of Nanomaterials and Nanosafety, Chinese Academy of Sciences Center for Excellence in Nanoscience, National Center for Nanoscience and Technology of China, Beijing, China; ^5^School of Nanoscience and Technology, Sino-Danish College, University of Chinese Academy of Sciences, Beijing, China; ^6^School of Chemical Engineering and Pharmacy, Wuhan Institute of Technology, Wuhan, China

**Keywords:** Alzheimer’s disease, mild cognitive impairment, artificial intelligence, predictive diagnostics, blood biomarkers

## Abstract

**Background:** There are no obvious clinical signs and symptoms in the early stages of Alzheimer’s disease (AD), and most patients usually have mild cognitive impairment (MCI) before diagnosis. Therefore, early diagnosis of AD is very critical. This paper mainly discusses the blood biomarkers of AD patients and uses machine learning methods to study the changes of blood transcriptome during the development of AD and to search for potential blood biomarkers for AD.

**Methods:** Individualized blood mRNA expression data of 711 patients were downloaded from the GEO database, including the control group (CON) (238 patients), MCI (189 patients), and AD (284 patients). Firstly, we analyzed the subcellular localization, protein types and enrichment pathways of the differentially expressed mRNAs in each group, and established an artificial intelligence individualized diagnostic model. Furthermore, the XCell tool was used to analyze the blood mRNA expression data and obtain blood cell composition and quantitative data. Ratio characteristics were established for mRNA and XCell data. Feature engineering operations such as collinearity and importance analysis were performed on all features to obtain the best feature solicitation. Finally, four machine learning algorithms, including linear support vector machine (SVM), Adaboost, random forest and artificial neural network, were used to model the optimal feature combinations and evaluate their classification performance in the test set.

**Results:** Through feature engineering screening, the best feature collection was obtained. Moreover, the artificial intelligence individualized diagnosis model established based on this method achieved a classification accuracy of 91.59% in the test set. The area under curve (AUC) of CON, MCI, and AD were 0.9746, 0.9536, and 0.9807, respectively.

**Conclusion:** The results of cell homeostasis analysis suggested that the homeostasis of Natural killer T cell (NKT) might be related to AD, and the homeostasis of Granulocyte macrophage progenitor (GMP) might be one of the reasons for AD.

## Introduction

Alzheimer’s disease (AD) is the most common chronic neurodegenerative disease (Burns and Iliffe, 2009). According to the World Health Organization, dementia affects 5–8 percent of people over 60 years. As of September 2020, there were about 50 million people with dementia, with 10 million new cases per year worldwide (Word Health Organization, 2020). Through establishing an individualized diagnosis model for patients with AD in its early onset, it is expected to realize early intervention for patients. At present, some studies have reported artificial intelligence models for AD diagnosis (Lunnon et al., 2013; Li et al., 2018; Way et al., 2018; Ludwig et al., 2019; Stamate et al., 2019). For example, in a European cohort study, a machine-learning approach identified 347 plasma metabolites associated with early diagnosis in AD with an area under curve (AUC) of about 0.85 (Stamate et al., 2019). In a study of circulating non-coding RNA in patients with AD, 21 disease-related features were identified using RT-qPCR, and 18 strongly correlated features were extracted using statistical learning methods to establish a machine learning model, with an AUC of about 0.86 (Herrero-Labrador et al., 2020). In an AD classifier based on texture features, the researchers modeled the high-level semantic features of MRI with an accuracy of about 85% (So et al., 2019).

However, these studies are based on the dichotomous task, ignoring the correlation degree of occurrence and development of the control (CON), mild cognitive impairment (MCI), and AD themselves, and the accuracy is not high. In this study, we incorporated blood mRNA expression data to establish two highly accurate artificial intelligence individualized diagnostic models for CON, MCI, and AD classification problems. Although a few studies analyze and/or predict these three disease states simultaneously, most of these studies are based on medical image data (Rogers et al., 2012). Furthermore, we analyzed the blood cells composition corresponding to blood mRNA profiles. We revealed some of the underlying mechanisms during the early pathogenesis of AD by analyzing the imbalance of five major groups of cells, including Epithelial, Hematopoietic stem cells (HSC), Lymphoid, Myeloid and Stroma. The overall landscape of blood cell imbalance lays a solid foundation for further mechanism research and individualized therapy.

## Materials and Methods

### Data Source and Preprocessing

We downloaded two sets of peripheral whole blood mRNA expression profiles from the GEO database^1^, including GSE63060 and GSE63061 (Sood et al., 2015). These two sets of data were detected by the platforms Illumina HumanHT-12 V3.0 and Illumina HumanHT-12 V4.0, respectively. After deleting fuzzy samples and finally keep 329 samples and 382 samples, a total of 711 samples (Table 1). We have carried out standardized processing in the quantity of the data set. The method is as follows: We mark the sample as *x*, and the expression value of the *j_th* gene in the sample as. First, calculate the sum of the expression values of all genes in the *i_th* sample, and then calculate the *j_th* gene in the *i_th* sample Divide by the sum in turn $\left(x_{i\,j}\,/\,\sum_{j=1}^{n}x_{i\,j}\right)$, and finally multiply the obtained value by 10^6^. The specific calculation formula is as follows:

xi⁢j′=(xi⁢j/∑j=1nxi⁢j)*10.6

**TABLE 1:** Data distribution diagram.

Datasets	Disease type	Sample numbers	Age [Median (Range)]	Sex (% male)
GSE63061	CON	104	73 (52−87)	40.38
	MCI	80	74 (63−90)	51.25
	AD	145	76 (58−88)	31.72
GSE63060	CON	134	74 (63−91)	39.55
	MCI	109	79 (57−100)	40.37
	AD	139	79 (59−95)	38.85

### Identification of Differentially Expressed Genes

The differential expression genes (DEGs) were recognized with the limma Bioconductor package (limma package v.3.24.15) in R (Smyth, 2005). The limma package use T-statistic as a discriminant that can eliminate the irrelevant genes. Limma package was use to findmarker by each two groups such as AD vs. CON, MCI vs. CON, and AD vs. MCI. We used the FDR-method correction for multiple testing.

### Feature Importance Selection

We use recursive feature elimination cross-validation to eliminate low importance. First, we choose a linear model to calculate all feature coefficients. Then we make a loop to eliminate low coefficient features until the number of features meets our requirements. This method is provided in the RFECV function, which is in the scikit-learn module in Python (Pedregosa et al., 2011; Buitinck et al., 2013). We use the default parameters of the RFECV function in the sklearn.feature_selection module, dependent variables are all genes, and independent variables are the results of the numerical transformation of CON, MCI, and AD set to 0, 1, and 2, respectively.

### Machine Learning Model

The machine learning models we use include linear models and non-linear models. Among them, the linear model uses a linear support vector machine (SVM) (Cortes and Vapnik, 1995), and the non-linear model includes AdaBoost (Freund and Schapire, 1997), random forest (Liaw and Wiener, 2002), and Artificial neural networks. Among them, linear SVM, random forest, AdaBoost use the function of the scikit-learn module of Python, Artificial neural networks use PyTorch module of Python. In the above four models, we use default parameters for the first three models; for Artificial neural networks, we use a feedforward neural network composed of three hidden layers and one output layer. The number of neurons in each hidden layer is 64, 32, and 16, respectively. The number of neurons in the output layer is 3, and the neurons in the output layer represent the probability values of various samples.

### XCell Analysis

XCell is a web analysis tool developed by the University of California, enriched based on gene expression data^2^ to obtain the Cell-Type score data (Aran et al., 2017). This method is based on gene signature, which is used to infer 64 types of immune cells and stromal cells.

### Disease Ontology Semantic and Enrichment Analysis

R (version 4.0.2) package DOSE (Yu et al., 2015) to analyze which diseases are related to the final features that we found. There are five functions in the DOSE package which we use is enriched function. Using cumulative hypergeometric model to identify which disease ontology that genes are mainly enriched in, where *k*is the number of genes related to the disease ontology; *r* is the number of all genes which are involved in all diseases that are collected in the DOSE package, and *s* is the number of genes which we have identified, the formula is as follows:

$$P=1-\sum_{i=0}^{s-1}\frac{\left(\begin{array}{c} k\\ i \end{array}\right)\,\left(\begin{array}{c} r-k\\ k-i \end{array}\right)}{\left(\begin{array}{c} k\\ r \end{array}\right)}$$

### Ingenuity Pathway Analysis

Ingenuity Pathway Analysis (IPA) is a bioinformatics analysis method. We use IPA method to locate features and annotate functions. *P*-value < 0.05 was considered a statistically significant threshold. *Z*-value greater than 0 is defined as active, and less than 0 is defined as suppressed. The activation z-score of a hypothesis is calculated from the regulation directions and gene expression changes of the genes in the overlap of data set and hypothesis-regulated genes. It assesses whether there is a significant pattern match between predicted and observed up- and down-regulation, and also predicts the activation state of the regulator (z > 0: activating, z < 0: inhibiting). The activation z-score is given by:

$$Z\,s\,c\,o\,r\,e=\frac{\left(N^{+}+N^{-}\right)}{\sqrt{N}}$$

with *N*^+^(*N*^–^) being the number of genes where the product of net-effect and observed direction of gene regulation is greater (less) than zero, and N = N++N^–^ (Krämer et al., 2014).

## Results

### Establishment and Analysis Process of Individualized Diagnosis Model for Overall Alzheimer’s Disease Patients

**FIGURE 1:**
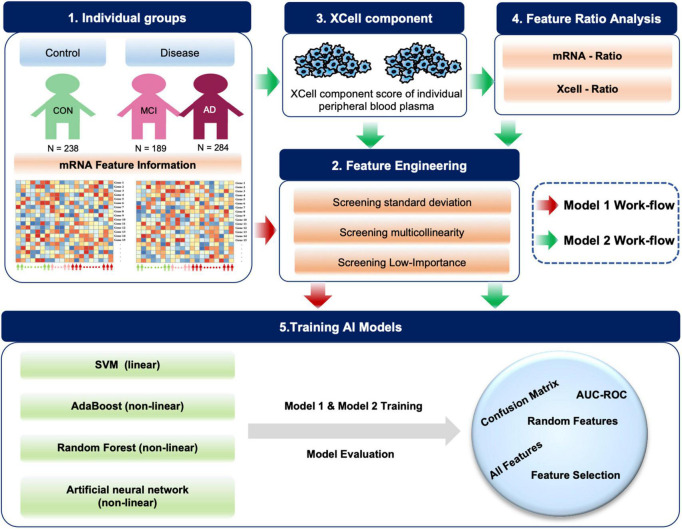
Diagram of the workflow in this study. A total of 711 individual peripheral blood mRNA data were included for modeling and analysis, including 238 control (CON), 189 MCI and 284 AD samples. For Model 1, we performed feature engineering, training and testing. For Model 2, XCell cell composition was first decomposed from blood mRNA data, then feature ratios were calculated using XCell and mRNA features, respectively. We combined mRNA, XCell, mRNA-ratio, and XCell-ratio to perform feature engineering. Four different algorithms were used for AI modeling. The models were evaluated on test data using AUC-ROC and confusion matrix. The red arrows represent Model 1, and the green arrows represent the processes for Model 2.

Based on blood mRNA expression profiles, we analyzed, screened and obtained two sets of potential blood biomarkers for early AD diagnosis and developed two different model frameworks (Figure 1). Individualized blood mRNA expression data of 711 patients were downloaded from the GEO database, including 238 CONs, 189 MCIs, and 284 AD patients. Firstly, we analyzed the subcellular localization, protein types and enrichment pathways of the differentially expressed mRNAs in each group, and established an artificial intelligence individualized diagnostic model. Furthermore, the XCell tool was used to analyze the blood mRNA expression data to obtain blood cell composition and quantitative data. New ratio features were established for mRNA and XCell data. Co-linearity and importance analysis of all features were carried out to obtain the optimal feature solicitation. Finally, four machine learning algorithms, including linear SVM, Adaboost, random forest and artificial neural network, were used to established models for the optimal feature set and evaluate their classification performance in test sets.

Next, we analyzed the effects of different mRNAs on the levels of different disease groups (MCI and AD) in terms of subcellular localization, coding protein type and enrichment function. First, we normalized the data and then identified 5,625 differentially expressed genes (DEGs) between the two groups (Supplementary Figure 1). Meanwhile, we performed cross-evaluation in different datasets. In addition, the results of the IPA show that DEGs in CON and MCI and DEGs in CON and AD differ not only in gene type but also in gene location. In the early stage of AD, the abundance of proteins located in the plasma membrane by DEGs is significantly up-regulated. In contrast, the expression of proteins located in other regions is inhibited. Specifically, the expression levels of Transmembrane receptor, G-protein coupled receptor, Phosphatase, and Kinase in the MCI group are increased. In addition, we also analyzed the same differential genes with disease-related enrichment and enriched and analyzed the up-regulated and down-regulated genes in the disease group (MCI and AD)/normal group, respectively. The upregulated genes are mainly enriched and associated with senile diseases. The down-regulated genes can significantly affect “Parkinson’s disease,” “Huntington’s disease,” “AD,” and “Oxidative phosphorylation.” NDUFA4, NDUFB6, ATP5F1C, CALM2, COX5B, COX4I1, and CYCS are also involved in the three major neurodegenerative diseases, including Parkinson’s disease, Huntington’s disease, and AD (Stelzer et al., 2016; Adav et al., 2019).

### Individualized Diagnostic AI Model Based on Blood mRNA Expression Data

**FIGURE 2:**
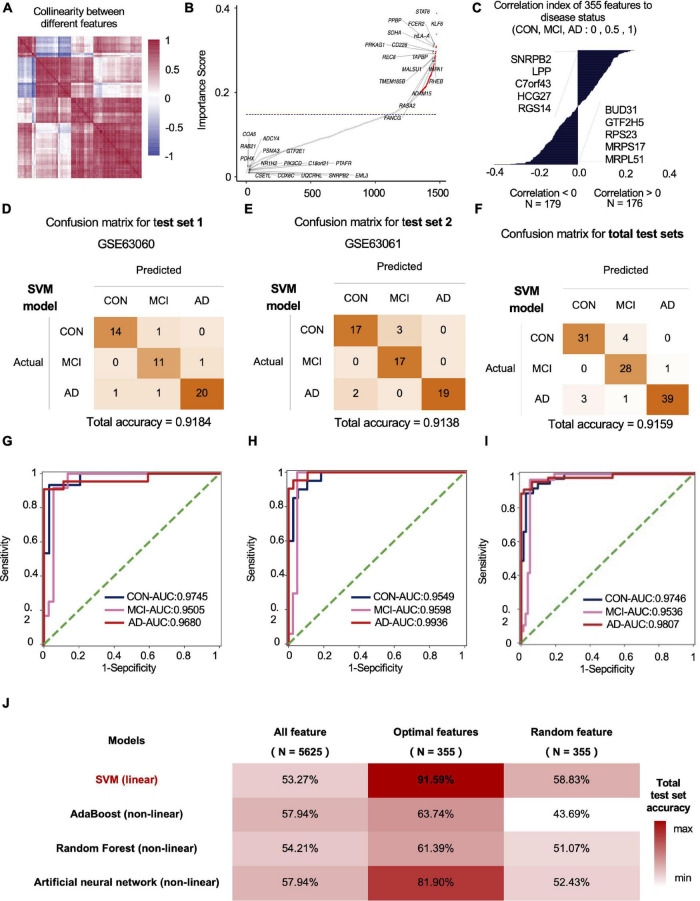
The mRNA data-based feature engineering and AI-modeling of CON, MCI and AD. **(A)** Correlation of each feature, correlation threshold 0.9. **(B)** Filter lower importance features, the y-axis represents the standard deviation, dashed line is the cutting threshold, the x-axis represents the features in order of importance (ascending). **(C)** Correlation between features and labels, show top five features. **(D–F)** Confusion matrix of two independent test sets. **(G–I)** ROC curve of the model developed by optimal feature in two independent test sets. **(J)** Compare the prediction accuracy on test set between different models and features.

We used standard deviation distribution, Co-linearity analysis (Figure 2A) and importance analysis (Figure 2B) to perform feature engineering screening on the total mRNA features for screening and to obtain the optimal feature set. In general, starting from 5,625 features, we screened out the features with a standard deviation less than 3 (retaining more than 75% of the features), and then the remaining 4,219 features. After the analysis of Co-linearity (Pearson correlation coefficient between various features is calculated), the features with a correlation greater than 0.9 are filtered out, leaving 1,598 remaining features. Importance analysis results showed that the TOP 5 with the highest contribution to tri-classification modeling are STAT6, KLF6, FCER2, HLA-A, PPBP, etc. (Figure 2B). We quantified the three states based on disease progression and assessed the correlation between the selected features and the disease state. Our results showed that SNRPB2, LPP, C7ORF43, HCG27, and RGS14 (Top-5) were positively correlated with the development of CON, MCI, and AD. Negative correlation features included BUD31, GTF2H5, RPS23, MRPS17, and MRPL51 (Top-5) (Figure 2C). After importance and correlation analysis (using an iterative method, removing 1% of the features in each iteration), optimal feature set was obtained for 355 mRNA features. We used the SVM algorithm to model the optimal feature set and then tested the model in two independent test sets, with the final test accuracy of 91.84 and 91.38%, respectively, and the average accuracy of 91.59% (Figures 2D–F). AUC values for CON, MCI and AD groups were 0.9746, 0.9536, and 0.9807, respectively (Figures 2G–I). Compared with the accuracy of optimal feature set (91.59%), the accuracy of SVM model established by all features (1,920 mRNAs) and random features (355 mRNAs) under SVM algorithm was 53.27 and 58.83%, respectively (Figure 2J). In addition to SVM, we also evaluate the classification performance of the models based on other algorithms. The accuracy rates of Adaboost, Random Forest and Artificial Neural Network test sets were 66.36, 62.62, and 80.56%, respectively, lower than the optimal model obtained by the SVM algorithm (Figure 2J).

### During the Progression of Alzheimer’s Disease, Both Blood mRNA and Blood Cells Showed Significant Expression Imbalance

**FIGURE 3:**
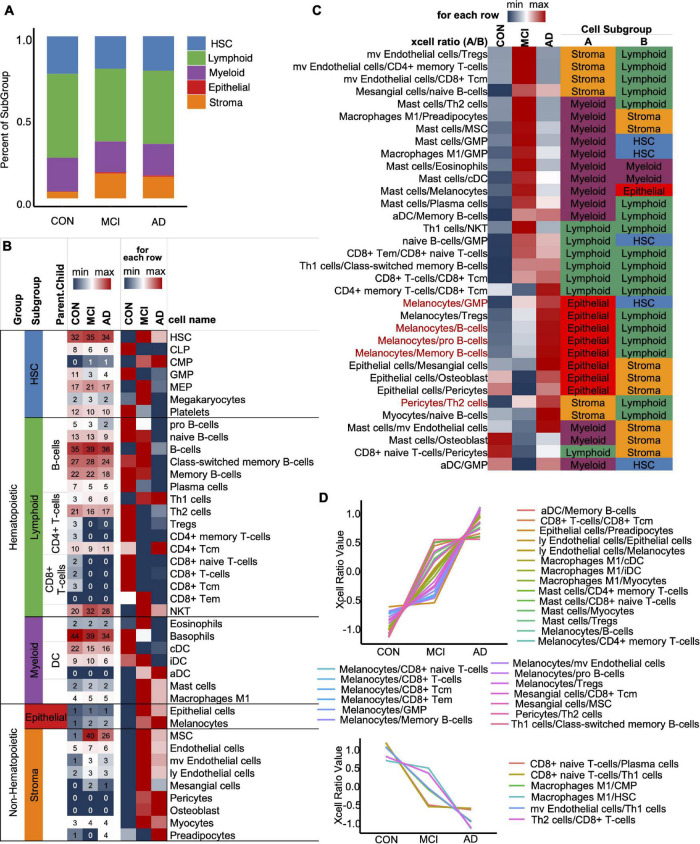
XCell and XCell-Ratio analysis. **(A)** Stacked five cell types in the CON, MCI, and AD groups. **(B)** Representative cells from five cell types. The number is original XCell score. **(C)** The union set of top-10 XCell-ratios in the con, MCI, and AD groups. All values are normalized by row. Red items, non-Hematopoietic/Hematopoietic items show a gradual trend with AD progression. **(D)** Cell ratios show a gradual trend with AD progression.

To optimize the classification efficiency of the artificial intelligence diagnostic model, we tried to include more biological information of different mRNAs. With the progression of AD, the composition of the various immune cells in the blood gradually changes. XCell is a method for inferring the quantitative abundance of 64 cell types based on mRNA expression data. We used XCell to analyze the quantitative level and composition of various cells in the blood and their changing trend with the progression of AD. Our results showed that the original blood mRNA expression profiles were mainly composed of five cell categories (41 cell subtypes), including HSC, Lymphoid, Myeloid, Epithelial, and Stroma. With the progression of AD, the proportions of HSC, Lymphoid and Myeloid in blood cells gradually decrease, while the proportions of Epithelial and Stroma gradually increase (Figure 3A). Figure 3B shows the absolute abundances of some representative cells (from all 41 cell types) and their relative abundances between the CON, MCI, and AD groups. To study the imbalance of cell proportion, we further analyzed the ratio of cell abundance. The top 10 cell ratios in CON, MCI, and AD groups were mainly related to immunity (58.8%). This result further suggest that AD disease is associated with immune dysregulation and can be recognized from the blood (Figure 3C; Ikeda et al., 2010). With the progression of the disease, we identified a total of 33 pairs of cells that showed a gradual change in the ratio. Most of them (27 pairs) gradually rise, including melanoma/GMP, melanoma/B-cells and melanoma/pro B-cells (Figure 3C, Red, Figure 3D, Up). The ratio of six pairs of cells decreased gradually, including CD8^+^ naive T-cells/Plasma cells and CD8^+^ naive T-cells/Th1 cells, etc. (Figure 3D, Down).

### Cell-Related Imbalance Can Be Included in the Feature Set to Participate in the Model Optimization

**FIGURE 4:**
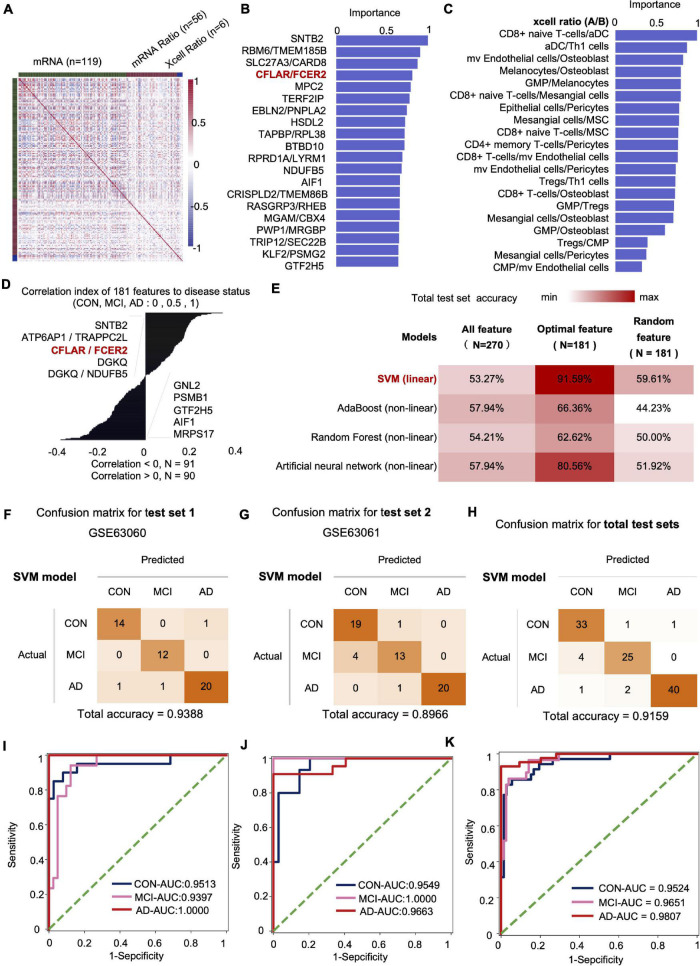
The mRNA and XCell ratio data-based feature engineering and AI-modeling of CON, MCI, and AD. **(A)** Feature type correlation heat map, an overview of the correlations between features after feature engineering. **(B)** Top 20 features importance (from optimal 181 features set). **(C)** XCell-Ratio of the top 20 in importance. The numbers represent the abundance of different cells in CON, MCI and AD. **(D)** The 181 features relevance between features and labels. **(E)** Compare the prediction accuracy on test set between different models and different features. **(F–H)** Confusion matrix of test set. **(I–K)** ROC curve of the SVM model developed by optimal features.

We fused four types of features, including mRNA, mRNA ratio, XCell, and XCell ratio for feature engineering to obtain the optimal feature set and subsequent AI modeling to diagnose CON, MCI, and AD (Figure 4A). Similar to the feature screening method in the first modeling, we recalculated the Co-linearity of each feature in the data, filtered out the remaining 956 features after the Co-linearity was greater than 0.9, and then eliminated the insignificant features by iterative method (each iteration removed 1% of the features), leaving 319. Finally, we selected the features with a cumulative weight greater than 75% to form a new optimal feature set. The optimal feature set contained 119 mRNAs, 56 mRNA-ratio pairs, and 6 XCell-ratio pairs. The mRNA ratio feature accounted for 60% of the top 20 importance rankings of the optimal feature set. Among them, the CFLAR/FCXER2 ratio with high importance was gradually increased in CON, MCI, and AD groups (Figure 4B). A previous study reported that the CFLAR is a vital gene encoding apoptosis regulator, and the FCXER2 is an important gene related to immunity (Stelzer et al., 2016).

The inclusion of XCell-ratio features reveals the importance of the imbalance of the proportion between different blood cells in the modeling of CON, MCI, and AD. The most essential XCell-ratio features for modeling included MV colorectal cells/Osteoblast, CD8^+^ naive T-cells/Mesangial cells and GMP/Osteoblast (Figure 4C). The optimal features are closely related to the progression of AD, where SNTB2, ATP6AP1/TRAPPC2L and CFLAR/FCER2 are positively correlated with the progression of AD. In contrast, MRPS17, AIF1 and GTF2H5 are negatively correlated with the progression of AD (Figure 4D).

### The Introduction of the Concept of Proportion Imbalance Is Beneficial to the Establishment of Artificial Intelligence Individualized Diagnosis Model

The imbalance of mRNA ratio and cell ratio was observed during the progression of AD. To evaluate the impact of the imbalance on AD diagnosis, we incorporated four algorithms, including linear SVM (linear model), Adaboost (non-linear model), random forest (non-linear model) and Artificial neural networks (non-linear model), to established artificial intelligence models. The results show that the accuracy of SVM algorithm is the highest. The accuracy of SVM, Adaboost, Random Forest and artificial neural networks for the test set were 91.59, 66.36, 62.62, and 80.56%, respectively. Compared with the optimal feature set, the accuracy of the model based on the total features and 181 random features was lower, indicating that our method of feature establishment, evaluation and screening is reasonable, effective, and reliable (Figure 4E).

At present, the accuracy of the best model obtained by the model in two independent test sets was 93.88 and 89.66%, respectively, with an average accuracy of 91.59% (Figures 4F–H). Notably, the average recall rate for AD patients in this set was 93.02%. Further analysis showed that the AUC values of the CON, MCI and AD groups were 0.9524 (CON and other groups), 0.9651 (MCI and other groups) and 0.9807 (AD and other groups), respectively (Figures 4I–K).

We also obtained a better tri-classification diagnosis model by including cell ratio and mRNA ratio data. Compared with the previously reported dichotomies, our model is more accurate and stable. Since we have comprehensively considered the changes in the occurrence and development of Con-MCI-AD, the model obtained in this study covers a wider area and applies to more potentially susceptible populations.

### Cell Ratio Analysis Showed That There Were Three Aspects of Immune Disorders During the Progression of Alzheimer’s Disease

**FIGURE 5:**
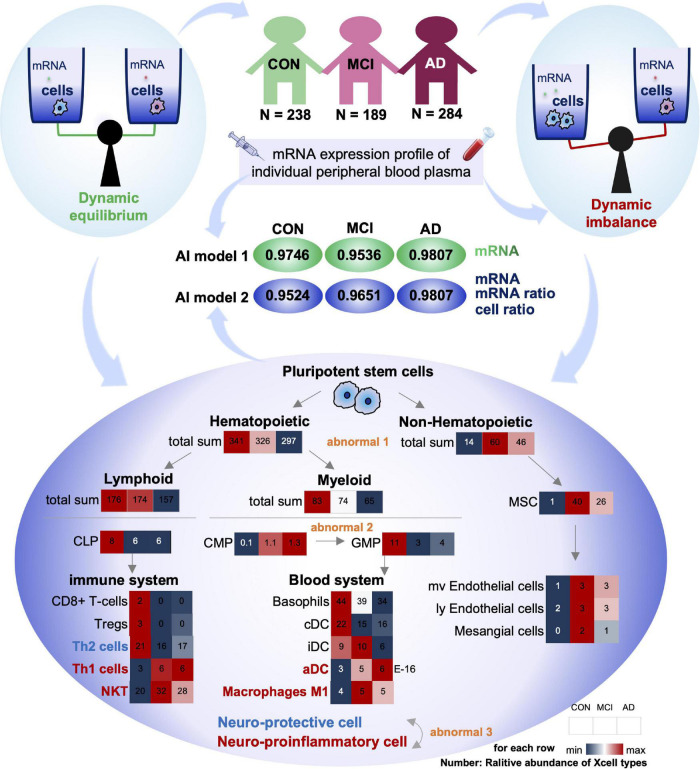
Summary of this study: dysregulation of cell homeostasis during the development of AD. Based on blood mRNA expression profiles, we screened two sets of deep learning models with AUC values greater than 0.95. There was a tripartite imbalance (orange color) in the relative abundance of blood cells in the patients, including a gradual decrease in the ratio of hematopoietic to non-hematopoietic cells, a block in the conversion from CMP to GMP, and a gradual decrease in the ratio of neuroprotective to neuroinflammatory cells. The values in the heat map represent the relative abundance of the XCell median for the disease. Our findings have important implications for both early diagnosis and intervention in MCI and AD.

To comprehensively analyze the imbalance of blood cell proportion in the body, we matched the variation trend of each cell with the progression of AD with the differentiation process of pluripotent stem cells. Our results showed three types of imbalances in the blood cells of patients with AD progression (Figure 5).

First, the proportion of hematopoietic and non-hematopoietic cells decreased gradually (Abnormal 1). The number of non-hematopoietic cell types in the disease group (MCI and AD) increased significantly compared with CON. MSC cells with multi-organ differentiation potential showed a more substantial increase in MCI patients than the CON. Its downstream, such as Endothelial cells and Mesangial cells, also showed similar trends. However, hematopoietic cell-related items were significantly downregulated in the disease group (MCI and AD). It has been reported that hematopoietic microglia can ameliorate the progression of AD by eliminating amyloid deposition through cell-specific phagocytic mechanisms (Lampron et al., 2011). Furthermore, hematopoietic cells and their associated factors have potential therapeutic value in AD, and the downregulation of the relative number of hematopoietic cells may be one of the critical theories (Sanchez-Ramos et al., 2008; Lim et al., 2020).

Secondly, the differentiation of CMP to GMP was resisted (Abnormal 2): in the hematopoietic cells, both Lymphoid and Myeloid cells showed a trend of gradual decrease. The Myeloid is the primary source of cells in the blood system. The common myeloid precursor cell index was significantly higher in the disease group (MCI and AD) compared with CON. As the downstream of its differentiation, the proportion of progenitor cells of granular macrophages decreased gradually. We speculated that blocking the differentiation of CMP to GMP might be closely related to the occurrence of MCI and AD.

Thirdly, the proportion of neuroprotective and neuroinflammatory cells was gradually reduced (Abnormal 3). Next, we analyzed the immune system differentiated by Lymphoid and Blood system differentiated by Myeloid. Our results showed that with the gradual development of AD, the immune system and circulatory system both changed from increasing to decreasing cell types. There is no apparent difference between the two systems. However, most of the progressively elevated cells are neuroinflammatory, including Th1, NTK, and ADC. The neuroprotective cells declined gradually, including Th2.

Compared with the CON group, the ratio of Th1 cells to Th2 cells in the disease group (MCI and AD) was significantly unbalanced. This conclusion is consistent with the results of previous animal immunotherapy experiments, which showed that Th1 cells decreased and Th2 cells increased in AD mice after immunotherapy (Town et al., 2002; Cao et al., 2009; Marciani, 2016). Notably, various indicators of NKT cells were similar to those of Th1 cells, suggesting that NKT cells also play an important regulatory role in the onset and progression of AD. However, no studies have confirmed that these cells play an important role in the onset and progression of AD. Therefore, NKT cells may be an important feature related to AD that has been recently discovered. In conclusion, we believe that the imbalance of homeostasis in GMP may be one of the important causes of AD, and the imbalance of homeostasis in NKT cells may be closely related to the occurrence and development of AD (Figure 5).

## Discussion

In this study, we established a complete feature engineering framework and an excellent machine learning model. We identified a set of features with stable classification efficiency based on the multidimensional data of mRNA expression profiles.

We found many mRNA features at the mRNA level that were significantly up- or down-regulated during AD progression. For example, the ATP5F1c gene is significantly down-regulated in the disease group (MCI and AD) compared to the CON group. The ATP5F1c was reported to play an important role in mitochondrial oxidative phosphorylation (Stelzer et al., 2016). The expressed level of mitochondrial electron transport chain complex IV (COX) was significantly reduced in AD patients. A previous study showed that genetic defects in the Cox family might be associated with the genetic risk of AD. In addition to the mRNA features, the mRNA ratio feature accounted for 60% of the top 20 importance rankings of the optimal feature set. Among them, CFLAR is an important gene encoding apoptosis regulator. FCXER2 is an important gene related to immunity (Stelzer et al., 2016). The importance of CFLAR/FCXER2 was higher, and its ratio gradually increased in CON, MCI and AD groups. We speculate that the occurrence and development of AD may be due to neuronal death caused by immune system abnormalities, and this process can be found in the blood.

At the cellular level, we also identified some particularly important features. For example, CD4^+^ and CD8^+^ (including CD8^+^ naive T cells, CD8^+^ T cells, and CD8^+^ TCM) cells were significantly lower in AD and patients with mild cognitive impairment. CD4^+^ T cells are effective mediators of well-known autoimmune diseases in the nervous system, such as multiple sclerosis and narcolepsy, which are involved in developing microglia (Pasciuto et al., 2020). Furthermore, we found a significant increase in the expression of myeloid cells represented by activated dendritic cells (ADC) in the disease group (MCI and AD). In contrast, the expression of lymphocytes represented by B cells was significantly reduced (Figure 3C). We found that many cells associated with the immune system showed a gradual increase during the occurrence and development of the disease (Figure 3D). Among the cell proportion features with noticeable progressive changes in expression, we found that almost all the proportion features were related to myeloid cells or lymphocytes (88.24%); among them, 58.8% were related to lymphocytes and 38.24% were related to myeloid cells.

We found that mitochondrial dysfunction in the brain tissue of AD patients can be simultaneously detected in the peripheral system (Johri and Beal, 2012), suggesting that AD may be caused by abnormal gene expression or brain damage, as observed in peripheral blood (Johri and Beal, 2012; Leuner et al., 2012; Trushina et al., 2013; Pérez et al., 2017). DEGs in peripheral blood may be one of the important causes of AD. Machine learning is an important branch of artificial intelligence. The main difference between this method and the traditional statistical learning method is that the machine learning method usually does not need a statistical hypothesis, which dramatically improves the accuracy of training results and the adaptability of the model, and is widely used in the study of the pathogenesis of AD (Farran et al., 2013; Goecks et al., 2020).

Compared with traditional feature engineering, this paper not only pays attention to feature selection but also pays attention to the development of new dimension features. In the cell type score obtained based on mRNA expression profile data, we found that Th and NKT cells were different between the disease group (MCI and AD) and the control group, with significantly fewer Th2 cells and significantly more Th1 cells and NKT (Figure 5). It is suggested that the occurrence and development of AD are closely related to immune system diseases, consistent with a previous report (Cui and Wan, 2019), we should pay high attention to the homeostatic dysregulation of NKT cells in AD. The features used in our model are highly interpretable.

## Conclusion

We find 5625 DEGs in CON, MCI, and AD, which are related to the disease. The optimal feature set was obtained through feature engineering screening, and the artificial intelligence individualized diagnosis model established based on this method achieved a classification accuracy of 91.59% in the test set. The AUC of CON, MCI, and AD were 0.9746, 0.9536, and 0.9807, respectively. The relative abundance of five types of cells, including Epithelial, HSC, Lymphoid, Myeloid and Stroma in the blood of CON, MCI and AD patients was obtained by mRNA expression profile analysis. We also included mRNA, cell abundance and ratio information to establish an artificial intelligence model. The diagnostic accuracy of the optimal model in the tri-classification test set was 91.59%, and the diagnostic AUC of CON, MCI and AD were 0.9524, 0.9651, and 0.9807, respectively. Based on the mRNA profiles, we analyzed the ratio of different cells using XCell. As patients progressively deteriorated from CON, MCI to AD, blood cells displayed three aspects of imbalance, including a progressive decrease in the proportion of hematopoietic cells, a block in the differentiation of CMP to GMP, and a progressive decrease in the proportion of neuroprotective/neuroinflammatory cells. Our findings have important implications for both early diagnosis and intervention in MCI and AD.

In this study, the composition of various cells in the blood of a single patient was analyzed based on the blood mRNA expression profile. Based on this, the balance between different mRNAs and cells in blood was analyzed. For the imbalance of disease and cell proportion in CON, MCI, and AD patients and their contribution to the artificial intelligence model, this study provides new ideas and results for the onset and progression of AD from both basic and application perspectives. The 181 features are composed of four dimensions, which can accurately classify CON, MCI, and AD groups, suggesting that machine learning methods can capture changes in blood biomarkers in AD patients. The results of cell homeostasis analysis suggested that the homeostasis of NKT cells might be related to AD, and the homeostasis of GMP might be one of the possible reasons for AD.

## Data Availability Statement

The original contributions presented in the study are included in the article/Supplementary Material, further inquiries can be directed to the corresponding author/s.

## Author Contributions

XZ and ZH designed the study. YS, BL, JZ, and YZ performed the analyses and interpreted the results. YS and XZ wrote the manuscript. XZ conducted this study. All authors read and approved the final manuscript.

## Conflict of Interest

The authors declare that the research was conducted in the absence of any commercial or financial relationships that could be construed as a potential conflict of interest.

## Publisher’s Note

All claims expressed in this article are solely those of the authors and do not necessarily represent those of their affiliated organizations, or those of the publisher, the editors and the reviewers. Any product that may be evaluated in this article, or claim that may be made by its manufacturer, is not guaranteed or endorsed by the publisher.

## Footnotes

1
https://www.ncbi.nlm.nih.gov/geo/

2
https://xcell.ucsf.edu/

## Supplementary Material

Supplementary Figure 1 The blood mRNA expression of CON, MCI and AD patients showed a different trend. **(A)** Heatmap of GSE63060 and GSE63061, rows represent genes, and columns represent samples. **(B)** DEGs of three groups between each other, bar-plot show the number of all up-down DEGs in different groups, the Venn-plot show that the intersection DEGs, the upset-plot show that all kind of intersections DEGs. The bar graph on the left represents the intersection of up-regulated differential genes between each group. In the middle of the scatter plot, the single point represents the unique gene in the data set, and the point-line-connection represents the intersection of the data and other data. **(C)** IPA localization and types score of DEGs, base-line (gray-line) CON/CON, red represent cell membrane localization. **(D)** Pathway enrichment results, the absolute value of x-axis is log10(*P*-value), the positive and negative signs represent up and down-regulation.

## Abbreviations

PD: Parkinson’s disease
AD: Alzhaimer’s disease
MCI: mild cognitive impairment
GEO: Gene Expression Omnibus
AUC: area under curve
IPA: ingenuity pathway analysis
DEGs: differentially expressed genes.
