# Supplementary figures and images for: Application of Artificial Intelligence Modeling Technology Based on Fluid Biopsy to Diagnose Alzheimer’s Disease
Source: Front Aging Neurosci. 2021 Dec 3;13:768229. doi: 10.3389/fnagi.2021.768229 (PMC8679840; doi:10.3389/fnagi.2021.768229)

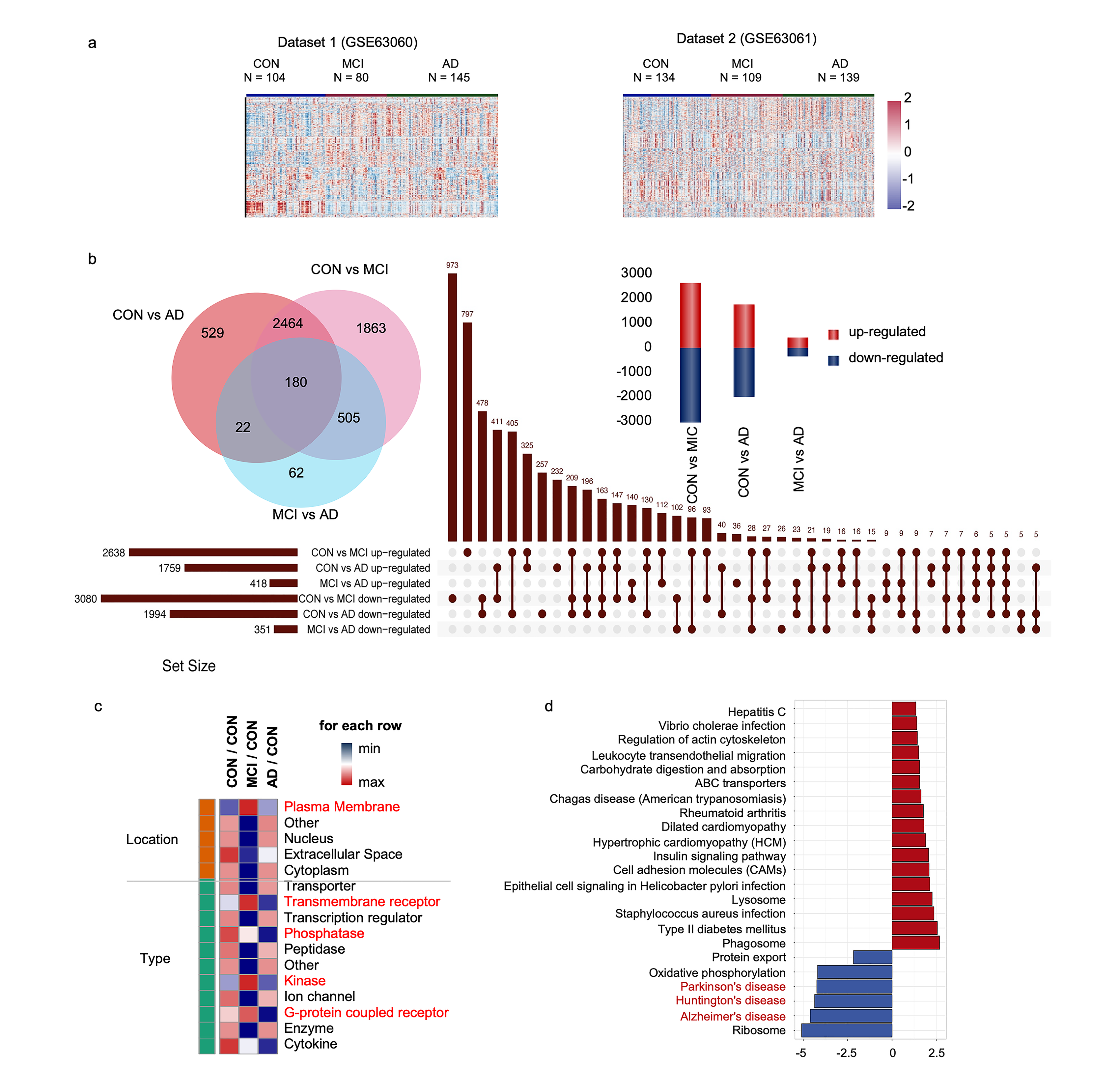

Supplement: Supplementary Figure 1 — The blood mRNA expression of CON, MCI and AD patients showed a different trend. (A) Heatmap of GSE63060 and GSE63061, rows represent genes, and columns represent samples. (B) DEGs of three groups between each other, bar-plot show the number of all up-down DEGs in different groups, the Venn-plot show that the intersection DEGs, the upset-plot show that all kind of intersections DEGs. The bar graph on the left represents the intersection of up-regulated differential genes between each group. In the middle of the scatter plot, the single point represents the unique gene in the data set, and the point-line-connection represents the intersection of the data and other data. (C) IPA localization and types score of DEGs, base-line (gray-line) CON/CON, red represent cell membrane localization. (D) Pathway enrichment results, the absolute value of x-axis is log10(P-value), the positive and negative signs represent up and down-regulation. [file Image_1.TIFF]
